# Designing and Fabricating Nano-Structured and Micro-Structured Radiation Shields for Protection against CBCT Exposure

**DOI:** 10.3390/ma13194371

**Published:** 2020-09-30

**Authors:** Kiana Nikeghbal, Zahra Zamanian, Shoaleh Shahidi, Gianrico Spagnuolo, Parisa Soltani

**Affiliations:** 1 Department of Oral and Maxillofacial Radiology, School of Dentistry, Shiraz University of Medical Sciences, Shiraz 71937, Iran; knikeghbal@yahoo.com; 2 Department of Oral and Maxillofacial Radiology, School of Dentistry, Shahrekord University of Medical Sciences, Shahrekord 88168, Iran; 3 Department of Occupational Health Engineering, School of Health, Shiraz University of Medical Sciences, Shiraz 71937, Iran; 4 Biomaterial Research Center, Department of Oral and Maxillofacial Radiology, School of Dentistry, Shiraz University of Medical Sciences, Shiraz 71937, Iran; shoalehshahidi@sums.ac.ir; 5 Department of Neurosciences, Reproductive and Odontostomatological Sciences, University of Naples “Federico II”, 80125 Naples, Italy; 6 Department of Oral and Maxillofacial Radiology, Dental Implants Research Center, Dental Research Institute, School of Dentistry, Isfahan University of Medical Sciences, Isfahan 81746, Iran; p.soltani@dnt.mui.ac.ir

**Keywords:** radiation protection, radiation safety, cone beam computed tomography, nanomaterial, nanostructured material, radiation dose

## Abstract

Researchers have always been interested in finding new and effective materials for protection against radiation. This experimental study aimed to design and fabricate new types of nano-material and micro-material based shields against the ionizing effect of cone beam computed tomography (CBCT) X-rays. To fabricate a flexible prototype, we added dioctyl phthalate (DOP) oil to emulsion polyvinyl chloride (PVC) powder. The paste was mixed and dispersed. Then, nano- and micro-powders of WO_3_ and Bi_2_O_3_ were added to the paste, with the weight ratio of 20% PVC, 20% DOP, and 60% nano- and micro-metals. Using an ultrasonic mixer, the polymer matrix and metals were mixed and a paste with a thick texture was developed. The resultant paste was poured into glass molds and the molds were then heated in an oven. After cooling, the resultant sheets were selected for further experiments. A CBCT unit and dosimeter were used to evaluate the characterization and X-ray shielding properties of the fabricated prototypes. The half-value layers (HVL) for nano-WO_3_, micro-WO_3_, nano-Bi_2_O_3,_ and micro-Bi_2_O_3_ were 0.0390, 0.0524, 0.0351, and 0.0374 cm, respectively. In addition, the linear attenuation coefficient (µ) for these materials were 17.77, 13.20, 19.71, and 18.5 cm^−1^, respectively. The findings indicate that nano-structured samples are more effective in the attenuation of X-ray energy. The nano-structured WO_3_ prototype was nearly 34% more efficient in attenuating radiation compared to the micro-structured WO_3_ prototype. This difference in nano- and micro-structured Bi_2_O_3_ prototypes was 6.5%.

## 1. Introduction

Radiological examinations are widely used for different diagnostic and treatment planning purposes in dentistry. Cone beam computed tomography (CBCT) is specifically focused on three-dimensional imaging of the maxillofacial region through data acquisition and image reconstruction. Fascination with CBCT in the field of dentistry is unprecedented, as has created a revolution in maxillofacial imaging, from diagnosis to image-guided operative and surgical procedures, by means of third-party software [1,2]. The use of dental radiography, however, is not without risk, since it exposes both patients and practitioners to ionizing radiation. Ionizing radiation is among the stimuli that induce production of free radicals, including cytotoxic monomers, tissue injury, inflammation, and physiological processes [3,4,5,6]. Free radicals, as well as direct DNA hits, result in DNA damage, which may in turn lead to deterministic and stochastic outcomes of radiation exposure [7,8,9]. CBCT imaging may generally expose the patients to higher radiation doses compared to other routine radiological examinations. In CBCT, based on field of view and other exposure settings, the dose increases to approximately 100 μSv, which is higher than other imaging modalities, such as panoramic, cephalometric, and intraoral radiographs [8,10,11]. According to the guidelines developed by the International Commission on Radiological Protection (ICRP), modern application of CBCT, and the associated radiation protection issues are markedly different from those of computed tomography. In many countries, dentists can equip their offices with CBCT units [12]. As a result of this, CBCT is now widely used by practitioners who have not received the traditional radiation safety training of specialists in radiology. Therefore, more emphasis on radiation protection equipment, guidelines, and standardization for CBCT procedures seems reasonable [13].

One of the simplest methods to reduce unnecessary radiation exposure, either to other organs of the body of the patient, or to the radiologist or technician is protective shielding. Due to its high atomic number and considerable density, lead has attracted much attention in X-ray shielding from the early days [14]. Lead can be used either in the form of lead sheets in walls and flexible lead aprons, or in conjunction with polymers. These aprons are rather heavy, and their long-term usage is not comfortable, and may even lead to back pain when frequently used [15]. Even though the lead aprons are flexible, they are fragile and prone to fracture [16]. This may in turn increase the probability of unwanted radiation exposure for individuals. In addition, lead is toxic and incompatible with the natural environment [17]. Lead aprons, when used and moved, can produce floating microparticles in air. These particles can be inhaled and cause lead intoxication. The permissible exposure limit for inhaled lead exposure is 50 µg in 1 m^3^ of air in 8 h [18]. Therefore, seeking substitutes for conventional lead protection shields is logical.

Over time, several metals, such as tin, tungsten, bismuth, etc. have been used as a substitute for lead in their pure or combined forms. Research continues on the substitution of lead aprons with lead-free ones [19,20]. According to recent research, it would appear that nanoparticles can induce increased protection against ionizing radiation. Metallic elements such as titanium, silver, zirconium, cobalt, barium, and lead have been used in nano-structured composites for radiation protection purposes [21,22,23]. It is clear that composites synthesized at the nano- and micro- levels will possess considerable flexibility due to their very small particle size, allowing them to be formed in various protective equipment [24]. This study aimed at fabricating and using new types of nano and micro WO_3_- and Bi_2_O_3_-based radiation shields against the harmful effects of CBCT X-rays.

## 2. Materials and Methods

In this experimental study, metal oxide nano-powders (60 nm for WO_3_ and 80 nm for Bi_2_O_3_) and micro-powders (4 µm for WO_3_ and 0.8 µm for Bi_2_O_3_), all with at least 99.5% purity (US Research Nanomaterials, Houston, TX, USA) were mixed in a polymer matrix to create nano or micro composites. Two sample shields of 2 mm ± 0.4 mm thickness were made for each material: 60 wt% of micro-WO_3_ in polymer matrix, 60 wt% of nano-WO_3_ in polymer matrix, 60 wt% of micro-Bi_2_O_3_ in polymer matrix, and 60 wt% of nano Bi_2_O_3_ in polymer matrix.

The density of each composition was calculated with the mathematical function 1/ρ = Ƹ Wfi/ρi, in which ρi is the density of metal-oxides, polyvinyl chloride (PVC) and dioctyl phthalate (DOP), while Wfi represents the weight percentage of these elements in the composition.

In order to prepare a polymer composition with respect to the 60% weight percentage, we used the ρ = m/v formula to calculate the mass of every component (Table 1). The volume was 7.6 cm × 7.6 cm × 0.2 cm for each sample.

**Figure 1:**
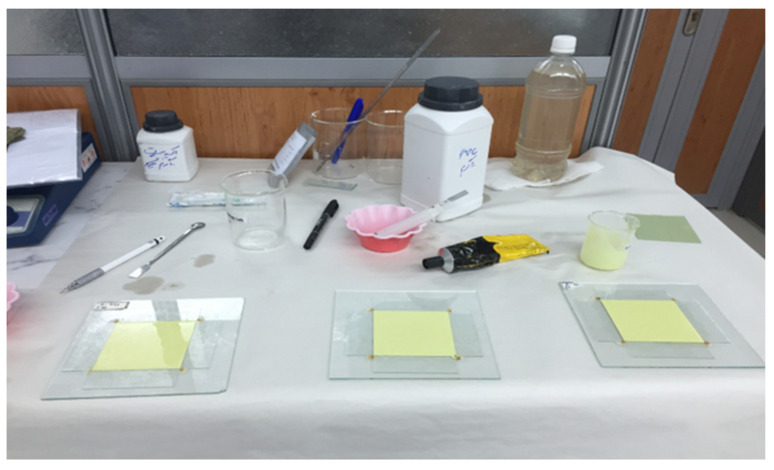
Preparation of tungsten and bismuth nano- and micro-structured prototypes.

To provide flexible radiation shields, the samples were made from flexible polymers. Emulsion PVC powder (LG Chem, Seoul, Korea) and DOP oil (LG Chem, Seoul, Korea), as softener, with equal weight proportions (5.3 gr for tungsten composite and 5.56 for bismuth composite) were poured into an ultrasonic mixer. The paste was mixed and dispersed for 15 min to become homogenous. Then, it was left for 6 h and mixed again before usage. At the time of use, the PVC particles were dissolved in the oil and could not be seen in the paste with a semi-liquid texture. Then, nano and micro powder of each metal element was added to the substance according to the mass quantities from Table 1. Using an ultrasonic mixer, the polymer matrix and micro- or nano-metals were mixed until a paste with thick texture was obtained. The resultant paste was poured into a glass mold with 7.6 cm × 7.6 cm dimensions and a depth of 2 mm, to get a homogeneous thickness. The filled mold was then heated for 45 min in an oven at 160 °C temperature. Thereafter, the mold was left at room temperature for at least 4 h. Then, the resultant solid sheets of the micro and nanocomposite prototypes (Figure 1) were tested for their flexibility and strength by manual handling, and two sample sheets were selected for each type of material (nano-WO_3_, micro-WO_3_, nano-Bi_2_O_3_, and micro-Bi_2_O_3_). For the characterization of synthesized tungsten and bismuth nano- and micro-structures, X-ray diffractometry (XRD) analysis was also used, using a D8 ADVANCE diffractometer (Bruker, Billerica, MA, USA) with a Cu anode. Powder XRD patterns were taken with steps of 0.02 at 1 s per step.

**Figure 2:**
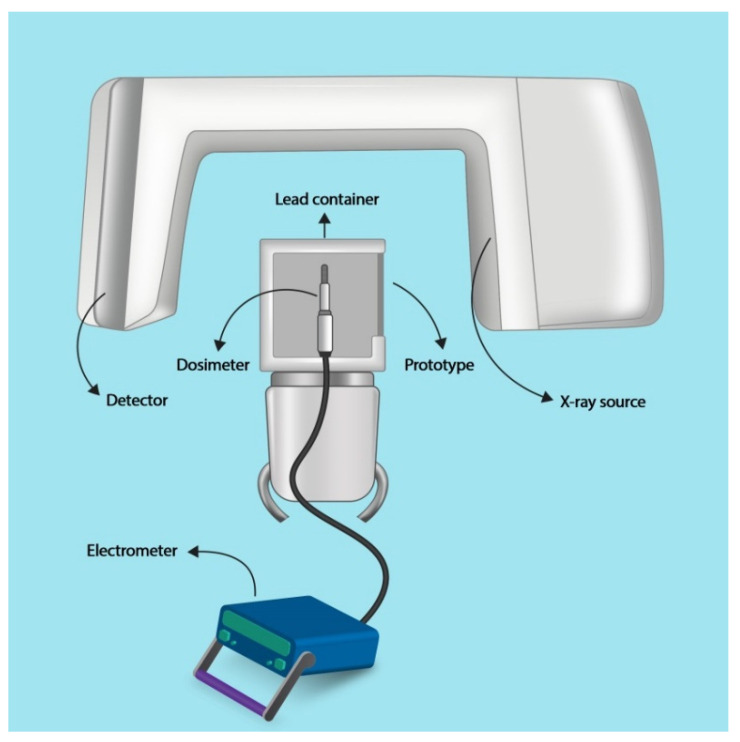
Schematic demonstration of the setup of the experiments.

To evaluate the characterization and X-ray shielding properties of the fabricated prototype, a CBCT unit (NewTom VGi, Verona, Italy) and a recently calibrated Farmer type ionizing chamber dosimeter (Scanditronix Wellhöfer, Schwarzenbruck, Germany) were used. The distance between the X-ray source and the sensitive area of the dosimeter was fixed in all measurements. The prototype plates were attached to a lead container covering the dosimeter. The area of the sheets and the lead container could completely obscure the sensitive area of the dosimeter. The dosimeter assembly was placed on the chin rest of the CBCT unit (Figure 2). Exposure factors were 110 KVP, 3 mA, and 1.8 s (pulsed), and a field of view of 15 × 15 cm was selected.

The intensity of CBCT radiation was measured three times without any absorbent shields (*I*ₒ). All three measurements showed the same value. Then, the first shield was placed in the dosimeter assembly and the passing intensity was measured (*I*). The passing intensity measurement was repeated three times for all of the prototype sheets.

Next, the linear attenuation index (µ) was calculated for each material, using the following formula (1):

(1) $$I=I_{0}e^{-µD}$$

In this equation, “*I*” represents the mean value for intensity of the passing rays for each shielding material, “*I*_0_” stands for the intensity of the primary rays, ”*D*” is the distance, and “µ” shows the linear attenuation index. In addition, the mass attenuation coefficient (µ/ρ) was calculated for each sample shield. For this, we had to calculate the density (ρ) of each constructed composite material by dividing their mass by their volume.

Having the value for the thickness of the samples, their half-value layer (HVL), i.e., the thickness of the protective material which reduces the intensity of radiation by one half, was calculated, using formula (2):

HVL = 0.693/µ(2)

Relative efficiency of HVL was calculated as below (3):

(3) $$\mathrm{Relative}\;\mathrm{efficiency}=\frac{\mathrm{HVL}\;\mathrm{nanomaterial}-\mathrm{HVL}\;\mathrm{micromaterial}}{\mathrm{HVL}\;\mathrm{nanomaterial}}\times 100$$

Finally, the measured intensities were plotted as charts using Statistical Package for the Social Sciences (SPSS, v.24, IBM, Armonk, NY, USA).

## 3. Results

### 3.1. Results of X-ray Diffractometry

**Figure 3:**
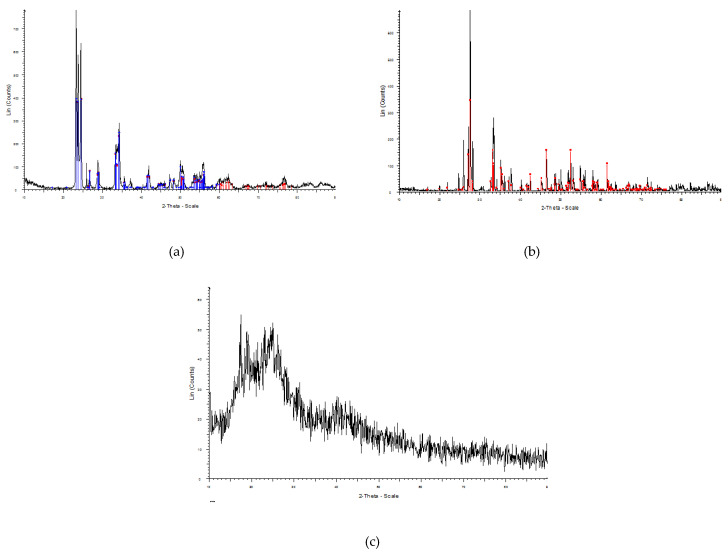
XRD pattern of: (**a**) WO_3_ nano powder; (**b**) Bi_2_O_3_ micro powder; (**c**) polyvinyl chloride (PVC) with the peaks similar to the reference patterns.

The peaks in the XRD pattern for synthesized tungsten and bismuth nano- and micro-structures were in excellent agreement with reference patterns for each material, showing the acceptable purity of the material (Figure 3).

### 3.2. Results of X-ray Attenuation Examinations

**Figure 4:**
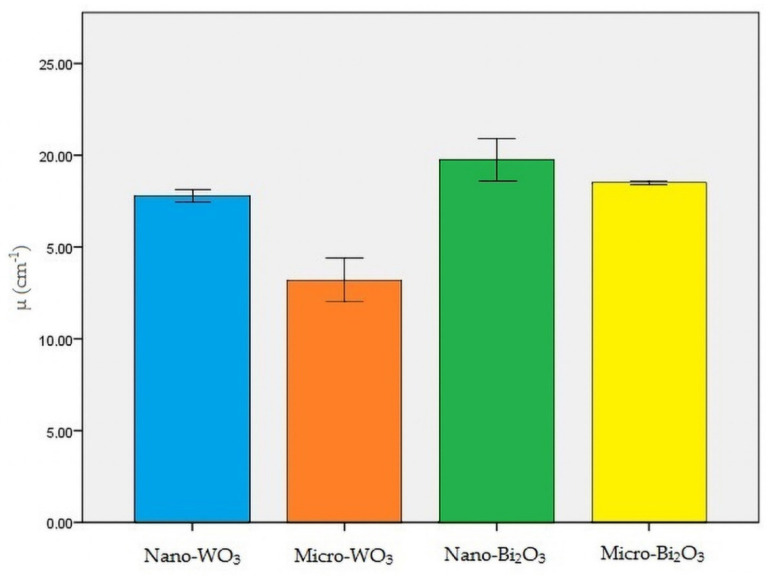
Linear attenuation coefficient (µ) of all the samples.

**Figure 5:**
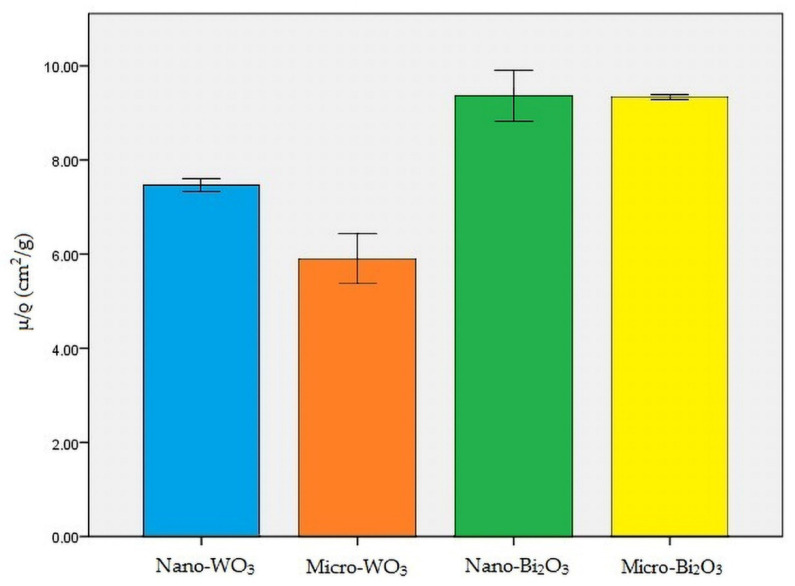
Mass attenuation coefficient (µ/ρ) of all the samples.

**Figure 6:**
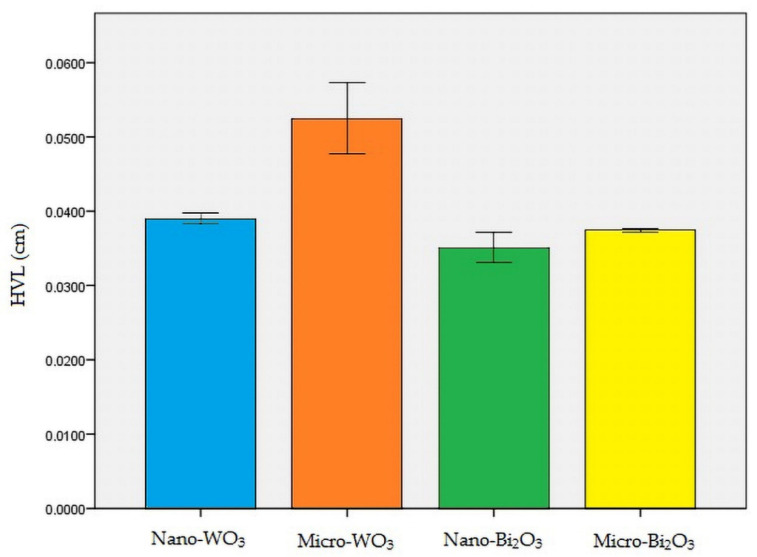
Half-value layer (HVL) of all the samples.

Nano-Bi_2_O_3_ showed the best shielding properties, followed by micro-Bi_2_O_3_. Nano-WO_3_ performed better in radiation attenuation compared to micro-WO_3_ (Table 2). Figure 4, Figure 5 and Figure 6 illustrate the linear attenuation coefficient, mass attenuation coefficient, and HVL of the fabricated prototypes.

According to the relative efficiency formula for HVL of the samples, nanostructured WO_3_ sample was nearly 34% more efficient in attenuating radiation compared to the micro-structured WO_3_ sample (formula 4). This difference in nano- and micro-structured Bi_2_O_3_ was 6.5% (formula 5).

(4) $$\mathrm{relative}\;\mathrm{efficiency}\;\left(\mathrm{HVL}\;{\mathrm{WO}}_{3}\right)=\frac{0.0524-0.0390}{0.0390}\times 100=34.3\%$$

(5) $$\mathrm{relative}\;\mathrm{efficiency}\;\left(\mathrm{HVL}\;{\mathrm{Bi}}_{2}O_{3}\right)=\frac{0.0374-0.0351}{0.0351}\times 100=6.5\%$$

## 4. Discussion

The present study aimed to test the X-ray absorption properties of four nano-structured and micro-structured radiation protection shields against CBCT exposure. Based on the results of the present study, HVL for nano-WO_3_, micro-WO_3_, nano-Bi_2_O_3_, and micro-Bi_2_O_3_ was 0.0390, 0.0524, 0.0351, and 0.0374 cm, respectively. In addition, the linear attenuation coefficient (µ) for the above-mentioned materials was 17.77, 13.20, 19.71, and 18.5 cm^−1^, respectively. Moreover, the mass attenuation coefficient (µ/ρ) for the above-mentioned materials was 7.46, 5.86, 9.354, and 9.351 cm^2^/g, respectively.

A simple way to reduce radiation exposure is the use of protection shields, which create a physical barrier between the X-ray device and individuals. These protection shields are conventionally made of lead and PVC, or other flexible polymeric materials. A thickness of 0.5 mm of equivalent lead is used for normal protection. Yet, the maximal protection is acquired with a thickness of 1 mm of equivalent lead at the expense of increasing weight. Several disadvantages of lead-based protective equipment, including heavy weight, toxicity, and susceptibility to cracks prompted researchers to seek new radiation protection substitutes.

Martinez and Cournoyer in their study, tested the radiation attenuation properties of tungsten, bismuth, copper, and iron [25]. They suggested that tungsten and bismuth can be used as non-hazardous substitutes for lead in radiation protection equipment. McCaffery et al. selected non-toxic metals with low atomic numbers (barium, tin, and antimony) and high atomic number (tungsten and bismuth) to use in a bi-layered designed apron [26]. The results of their study showed superior attenuation properties of the novel designed bi-layered apron in comparison with the conventional lead apron. Nano-structured composites have also been studied as radiation protection material against harmful effects of ionizing radiation in unwanted areas of the body. Ayyildiz et al. tested titanium, zirconium, silver, and cobalt nanocomposite materials for use as radiation protection shields [23]. They revealed that cobalt nanocomposite can be used as a shielding material against the harmful effects of X-rays during diagnostic dental radiography or radiotherapy. Moreover, Nambiar et al. examined the applicability of polydimethyl-siloxane-nanocomposite protection shields against radiation [24]. They reported that this nanocomposite possesses the potential to effectively attenuate X-rays (primary and phantom-scattered) generated during interventional radiography procedures, so that it can be considered as a protective material during interventional radiography procedures. Jiang et al. in their study, prepared a sample of BaSO_4_/cellulose nanocomposite and proposed its further application for radiation protection [21]. In addition, Vagheian et al. fabricated nano-structured thin lead sheets. They concluded that for low-energy X-rays, nano-structured material attenuate more photons than bulk-structured samples [22].

In order to choose a substitute for lead, the authors reviewed the current literature. Tungsten was chosen due to its high density and bismuth was selected because its atomic number is higher than that of lead. Tungsten and bismuth have been previously used for manufacturing novel radiation protection shields. For example, Aral et al. used cotton fabrics coated with silicone rubber containing tungsten and bismuth. They concluded that these novel radiation shields can be useful in protection against X-ray energy [27]. In addition, the applicability of nano-WO_3_, micro-WO_3_, nano-Bi_2_O_3_, and micro-Bi_2_O_3_ as X-ray shielding material is mentioned in the literature [28,29,30,31].

Although the HVL of lead for 110 KV X-ray (nearly 0.300 cm) is less than all nano- and micro-structured shields, nano-WO_3_ or nano-Bi_2_O_3_ protection shields, which provide equal X-ray attenuation to lead, are still much lighter than the lead shield. Thus, it can be concluded that novel shielding materials can be more comfortable compared with conventional lead aprons.

The findings of this study indicate that under CBCT exposure, nano-structured samples are more effective in the attenuation of X-ray energy. Azman et al. reported that nano-sized WO_3_ composite is more effective than micro-sized WO_3_ composite in radiation protection [28]. A similar trend was also observed in the study by Botelho et al. According to their results, CuO nanocomposite exhibited better attenuation characteristics compared with CuO micro-composite [32]. In an equal mass of nano- and micro-structured material, the number of nanoparticles is higher than microparticles, with a higher surface-to-volume ratio. Eventually, nano-structured composites consist of a higher number of particles per gram when compared with micro-structured composites. Moreover, as the particle size is reduced, the number of superficial atoms is increased; thus, some atomic linkages are broken, providing free electrons. This increases the electron cloud mass of free electrons in nanostructured material. Moreover, nano-sized particles are more uniformly distributed in the polymeric matrix. This leads to the increased probability of interaction of the X-ray with metal atoms and free electrons in nano-structured shielding material. Therefore, nano-structured shielding materials can be lighter than micro-structured ones, as well as providing equal radiation attenuation.

To the best of our knowledge, this is the first study to examine the radiation protection characteristics of nano- and micro-structured WO_3_ and Bi_2_O_3_ against CBCT exposure. CBCT was used as it is the modality with the highest radiation dose in the field of oral and maxillofacial radiology. Moreover, the geometry of the beam and scan parameters are different from computed tomography or conventional radiographs. The findings of the present study are helpful for developing a novel radiation protection apron for usage in the practice of oral and maxillofacial imaging.

One of the limitations of the present study was that we performed the experiments using 60 wt% of the nano- and micro-metals, and were unable to repeat the experiments with different weight percentages due to financial constraints. Another limitation of the study is that in practical scenarios, the geometry of source, object and detector may be different from the experimental conditions. In addition, scattered secondary rays are present when the patient is exposed, which can lead to obtaining different results in experiments and practical patient exposure. The authors tried to control the sources of error in this study. For instance, the masses of the material used for fabrication of the prototypes were measured using a scale with a sensitivity of 0.01 gr. However, these masses might have insignificant differences with each other and with the intended values. Furthermore, we measured the distance between the prototype sheets and the X-ray source for each experiment with 1 mm sensitivity, and a negligible variation may exist in this distance among the experiments. Nevertheless, we performed the experiments for each prototype three times and also used two prototype sheets for each shielding material to account for these errors. It is important to note that these nano-structured material can be coated or painted, and can practically conform to any shape of interest. Therefore, they can be applied in construction of different radiation protection equipment such as thyroid shields and protective aprons or gloves. With the advancements in nanotechnology, the current trend is toward exploiting the properties of nanostructured material in order to create advanced nanocomposites for effective, light-weight, and durable radiation-resistant equipment.

## 5. Conclusions

Nano-structured composites were generally more effective than micro-structured composites in attenuation of CBCT radiation. The prepared products can be considered as light-weight and non-toxic radiation shielding materials, with a wide range of capabilities in the fabrication of protective equipment.

## Figures and Tables

**Table 1:** Calculated composite component mass.

60 wt% Metal Oxide Powder (Micro or Nano)	20 wt% PVC	20 wt% DOP
16.08 gr WO_3_	5.3 gr	5.3 gr
16.70 gr Bi_2_O_3_	5.56 gr	5.56 gr

**Table 2:** Characteristics and X-ray shielding properties of the materials.

Material	Ρ (g/cm^3^)	D (m)	I_0_ (photons/cm^2^)	I (SD) (photons/cm^2^)	I/I_0_	µ (cm^−1^)	µ/ρ (cm^2^/g)	Half-Value Layer (HVL) (cm)
Nano-WO_3_	2.380	0.2	2.24 × 10^2^	6.408 (0.14)	2.86 × 10^−2^	17.77	7.46	0.0390
Micro-WO_3_	2.236	0.2	2.24 × 10^2^	15.985 (1.27)	7.136 × 10^−2^	13.20	5.86	0.0524
Nano-Bi_2_O_3_	2.108	0.2	2.24 × 10^2^	4.327 (0.32)	1.94 × 10^−2^	19.71	9.354	0.0351
Micro-Bi_2_O_3_	1.980	0.2	2.24 × 10^2^	5.527 (0.03)	2.467 × 10^−2^	18.51	9.351	0.0374
